# Time and energy costs of different foraging choices in an avian generalist species

**DOI:** 10.1186/s40462-019-0188-y

**Published:** 2019-12-30

**Authors:** Alejandro Sotillo, Jan M. Baert, Wendt Müller, Eric W. M. Stienen, Amadeu M. V. M. Soares, Luc Lens

**Affiliations:** 1 Department of Biology, Terrestrial Ecology Unit, Ghent University, K.L. Ledeganckstraat 35, 9000 Ghent, Belgium; 2 Department of Biology & CESAM - Centre for Environmental and Marine Studies, University of Aveiro, Campus de Santiago, 3810-193 Aveiro, Portugal; 3 Department of Biology – Behavioural Ecology and Ecophysiology Group, University of Antwerp, Campus Drie Eiken, Universiteitsplein 1, 2610 Wilrijk, Antwerp Belgium; 4 Research Institute for Nature and Forest (INBO), Herman Teirlinckgebouw, Havenlaan 88, 1000 Brussels, Belgium

**Keywords:** Energy expenditure, Foraging, Central place, GPS tracking, ODBA

## Abstract

**Background:**

Animals can obtain a higher foraging yield by optimizing energy expenditure or minimizing time costs. In this study, we assessed how individual variation in the relative use of marine and terrestrial foraging habitats relates to differences in the energy and time investments of an avian generalistic feeder (the Lesser Black-backed Gull, *Larus fuscus*), and how this changes during the course of the chick-rearing period.

**Methods:**

We analyzed 5 years of GPS tracking data collected at the colony of Zeebrugge (Belgium). Cost proxies for energy expenditure (overall dynamic body acceleration) and time costs (trip durations and time spent away from the colony), together with trip frequency, were analyzed against the relative use of the marine and terrestrial habitats.

**Results:**

The marine habitat was most often used by males and outside weekends, when fisheries are active. Marine trips implied higher energetic costs and lower time investments. As chicks became older, terrestrial trips became more prevalent, and trip frequency reached a peak towards 20 days after hatching of the first egg. Over a full chick rearing period, energy costs varied widely between individuals, but no trends were found across the marine foraging gradient. Conversely, a higher use of marine foraging implied lower overall amounts of time spent away from the colony.

**Conclusions:**

Foraging habitat choice was related to overall time costs incurred by gulls, but not to energy costs. The effect of chick age on foraging habitat choice and effort may be driven by energy expenditure constraints on the amount of marine foraging that can be performed. If time is less constraining to them, Lesser Black-backed Gulls may meet the increasing chick demand for food by switching from high to low energy demanding foraging strategies.

## Background

Time and energy constitute key currencies in animal ecology, and their efficient use is a primary criterion for natural selection [1–4]. Costs and benefits of behavioural traits are therefore often evaluated in terms of both currencies [5–8]. In the context of foraging behavior, Optimal Foraging Theory [9] predicts animals to maximize their net energy intake per unit of time investment [10], in particular when individuals are on a tight time budget and face energetic constraints, such as when provisioning food to their developing young [11]. Food provisioning strategies that result in a higher yield for a given time or energy cost can on this basis be expected to result in higher reproductive success.

Yet, the pronounced individual variation in foraging strategies observed in many animal populations [12–14], suggests that the relative costs and/or benefits of different strategies may vary with intrinsic factors [15]. These factors include morphology [16, 17], sex and age [18–20], as well as personality [21, 22]. Changes in extrinsic factors, such as environmental conditions, can alter the availability of particular food sources and the costs involved in their use [23–25], thus affecting the adaptive value of different foraging strategies and, consequently, the adaptiveness of the individuals displaying them. Assessing the trends in food resource use, and the time and energy costs in relation to them, can help predict how future environmental changes may affect foraging efficiency, and whether some individuals in a population might be more impacted than others by such changes.

Some opportunistic species have recently adapted to using anthropogenic food sources, which are considered to buffer or substitute the natural variation in resource availability [26–29]. Given the stability of these human food subsidies, animal populations relying on them provide a convenient model to compare costs between the use of different food sources and individual strategies. Recent improvements in tracking technology have increased the accuracy at which costs related to foraging behavior can be assessed [30–33]. For instance, tri-axial accelerometers provide data on the fine-scale body movements of an animal, which can be integrated to obtain proxies for energy expenditure, such as the overall dynamic body acceleration –ODBA [34, 35].

Here, we focus on the Lesser Black-backed Gull (*Larus fuscus*), an opportunistic seabird species with strong individual variation in foraging specialization [36–38]. In gulls of the genus *Larus*, the diversity in foraging behavior within a breeding population depends on the foraging opportunities around the colony [14, 38–41]. Since the mid-twentieth century, Lesser Black-backed Gulls increasingly depend on human activities: at sea they largely rely on fishery discards [42–44], while on land, garbage in cities and soil organisms at agricultural fields form their main food sources [45–47]. This renders Lesser Black-backed Gulls –depending on the foraging preference- vulnerable to changes both in weather variables and in human activities [38]. To properly predict and mitigate the effects of environmental changes, it is important to gain more insight into the variation in use and efficiency of different foraging strategies at the individual level.

To achieve this aim, we analyzed movement behavior of adult Lesser Black-backed Gulls breeding in the coastal colony of Zeebrugge (Belgium) during chick food provisioning. We assessed whether, and to what extent, time- and energy costs vary between marine and terrestrial foraging. Marine fish have often been assumed to be the preferred chick diet for this species, given the ubiquity of this resource use by Lesser Black-backed Gulls in the North Sea [48–50] and its positive effects on chick growth and survival [51, 52]. However, given the highly competitive conditions to obtain food at fishing vessels [53, 54], we expect marine foraging to imply higher costs. Based on high-resolution GPS-tracking data, we determined each individual’s relative use of marine and terrestrial feeding grounds, and calculated cost proxies for energy (ODBA) and time (time spent away from the colony and trip duration), as well as trip frequencies. We expected foraging investments to increase with advancing chick age in order to meet the growing chicks’ demand for food [55], and thus further assessed the effect of chick age on the prevalence, time and energy costs of marine and terrestrial foraging, calculated on a foraging trip basis and per day.

## Methods

### Satellite tracking

Between 2013 and 2018, a total of 75 breeding adult (i.e. at least 4 years of age) Lesser Black-backed Gulls were equipped with UvA-BiTS GPS tracking devices [56, 57] in the Port of Zeebrugge (51°20′53″N 3°10′20″E), which hosted between 1181 and 3331 breeding pairs during this period. Only data from the chick rearing period were used for analysis, extending from the hatching date of the first chick until the youngest chick was 30 +/− 2 days of age, unless chicks died before. Brood size was standardized at 2 chicks per nest, which were cross-fostered: the original eggs were substituted by 2 pipping eggs obtained from 2 different, haphazardly chosen nests, selecting only first- or second- laid eggs. This procedure standardized offspring demand, promoted hatching synchrony within broods, and removed parental genetic effects on chick growth and survival in the context of a study on potential effects of tagging on breeding performance [58]. If age nevertheless differed between chicks within broods, the age of the oldest chick was used for statistical analysis. To avoid biases in the calculations of cost proxies due to insufficient data, day-based cost proxies were calculated for adult individuals with more than 15 full days of tracking data available during the chick rearing period (*N* = 66, Additional file 1: Table S1). Of these, 33 individuals had 10-s samples containing 200 accelerometer measurements coupled to each GPS position, used for the calculation of ODBA.

GPS trackers were installed during incubation, when gulls can be trapped by means of a walk-in trap placed on the nest. Position data was collected at different resolutions depending on the year and location inside or outside of a 2.4 km^2^ area delimiting the nesting colony (Additional file 1: Figure S1): a fix was recorded every 1, 2 or 3 min outside of the colony, and every 15, 20 or 30 min inside the colony. To avoid biases in the calculation of derived variables arising from the differences in temporal resolution, location data were resampled to a 3 min resolution for trip-based calculations and to a 30 min resolution for day-based calculations.

### Proportion of marine trips

A foraging trip comprised all activities, including both moving and resting behaviors, performed in the time between leaving the colony and returning to it, using as reference a buffer of 10 Km radius from the center of the colony (see Additional file 1: Figure S1), and was assigned to the day when it was initiated. The 10 Km buffer excludes resting areas in the vicinity of the colony, including the whole port area, where foraging rarely occurs. This resulted in a sample of 2964 trips in Zeebrugge. Given that the proportions of marine fixes per trip concentrate at values below 10% (mostly positions recorded when flying over the sea to and from the colony) and above 90% (Additional file 1: Figure S2), a foraging trip was labelled as “marine” if at least 90% of its fixes were recorded at sea. Per individual and day, and also for a full chick rearing period, the proportion of marine trips over the total number of trips was calculated.

### Cost proxies

Per trip, we calculated mean ODBA as a proxy of energy expenditure rate (as in e.g. [34, 59, 60]), and trip duration (h) as a measure of time investment. On a daily basis, the number of trips initiated, the sum of ODBA as proxy for the daily energy expenditure (as in e.g. [61–63]), and the total time spent away from the colony were calculated per individual. Over a full chick-rearing period, and per individual, averages were calculated for: the daily sums of ODBA, the daily time spent away from the colony (average daily time spent foraging), trip durations, and the number of trips initiated per day (trip frequency). Day-based calculations were made on days containing at least 43 location fixes at a 30 min resolution (thus covering at least 90% of the full 24 h cycle, Additional file 1: Figure S3). When calculating averages over a full chick rearing period, daily values were weighted to account for missing data.

ODBA was used as a proxy for energy expenditure [35] in individuals for which accelerometer data was available (Additional file 1: Figure S4). Tri-axial acceleration measurements were converted into units of *g* (1 *g* = 9.8 m s^− 2^), by subtracting the device’s offset and dividing by its sensitivity for the corresponding axis. For each GPS fix with an associated accelerometer sample (20 Hz for 10 s = 200 measurements per sample), the sum of ODBA in the x, y and z directions was obtained as:

$$ ODBA=\mid DAx\mid +\mid DAy\mid +\mid DAz\mid $$

Where DA is the mean dynamic acceleration component (due to the animal’s movement) along the x, y and z axis, obtained by subtracting the estimate for static acceleration (due to the Earth’s gravitational field) from the corresponding accelerometer measurement. Static acceleration was approximated as the running mean over the full 10 s sample of all measurements along each axis.

Mean ODBA was calculated for trips where at least 90% of fixes had accelerometer measurements (*N* = 1633; 55% of the original sample), and summed daily ODBA were calculated for days where at least 90% of fixes had accelerometer measurements (*N* = 937 days; 61% of the original sample). We assumed ODBA to accurately reflect the energy expenditure rates (mean trip ODBA) and daily energy expenditure (summed daily ODBA) in our sample, given its direct relationship at both temporal scales with the amount of flapping flight (Additional file 1: Figure S5), which has elsewhere been used as a proxy for energy expenditures [64], based on the assumption that it is the most energetically expensive form of locomotion [65].

### Data analysis

Variation in the probability for a trip to be marine was tested by means of a binomial generalized linear mixed model (GLMM) with a logit link function, where the birds’ sex, chick age and a factor discriminating weekends from weekdays (to account for the lack of fisheries activity during weekends) were included as explanatory variables, as well as all relevant interactions. Year and bird identity nested within year were included as random intercepts.

The trip-based variables (mean trip ODBA and trip duration) were analyzed by means of linear mixed effects models, against chick age, a factor discriminating between marine and terrestrial trips, and their interaction. Year and bird identity nested within year, were included as random intercepts. In addition, a first order autocorrelation structure was fitted.

The summed daily ODBA and time spent away from the colony were analyzed against chick age in the same way as the trip-based variables, but since birds could perform both marine and terrestrial trips in a same day, a daily proportion of marine trips was used instead of the factor discriminating between marine and terrestrial trips, and the time covariate was chick age. Sex was included as a covariate in all trip- and day-based analyses of cost proxies, but no significant effect was found in any case (Additional file 1: Table S2). Consequently, this variable was removed from the final models.

The daily number of trips initiated was regressed on chick age as a second degree polynomial, including sex as a covariate, by running a zero-inflated count data regression. A zero-inflated model was chosen given the fact that in 19% of individual-days no trips were performed. At the scale of the full chick rearing period, the average cost proxies were analyzed against the overall proportion of marine trips per individual, by means of linear regression.

For the linear mixed effects models and GLMMs, significance of the model terms was tested by means of an analysis of deviance between the full model and a model without the corresponding term, using type III Wald Chi-squared tests. For linear regressions, a type III analysis of variance was performed instead, using F-tests. All statistical analyses and figures were produced in R [66] (Additional file 2). Linear mixed effects models and GLMMs were built using package lme4 [67] and tested using package lmerTest [68]. The Poisson regression for zero-inflated data was performed using package pscl [69]. Estimated marginal means and factor coefficients were obtained using package emmeans [70]. The significance level of all performed tests was set at 5%.

## Results

**Fig. 1:**
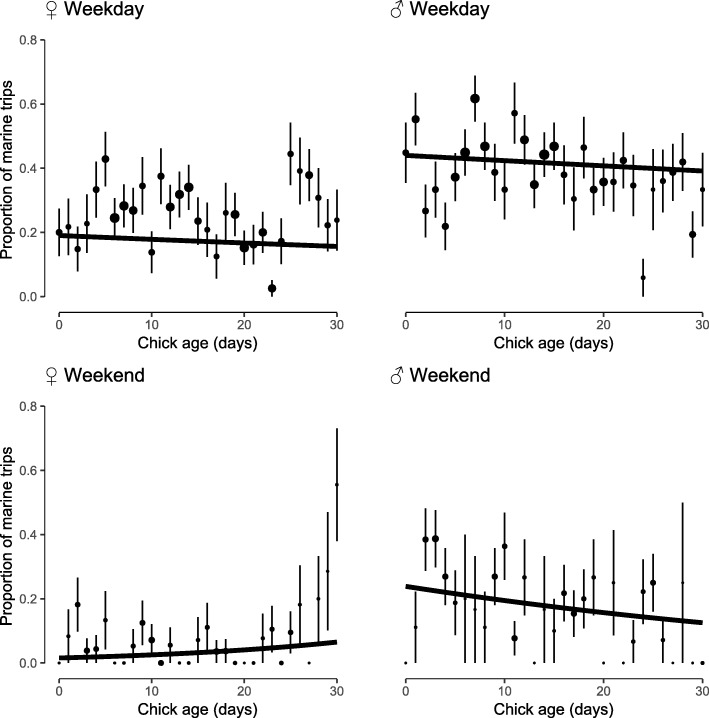
Average proportion of marine trips performed by gulls, against the number of days after hatching of their first egg, plotted separately by sex and between working days and weekends. Bars indicate the standard error of the mean. Lines are plotted for the significant relationship between the proportion of marine foraging trips and the number of days after hatching of the first egg, per combination of sex and period of the week. Predicted values are back-transformed from the logit scale. Size of points is proportional to sample size

The total percentages of marine trips per individual ranged between 0% (fully terrestrial foragers, 5 females) and 97% (almost fully marine foragers, 1 male), with a median of 21% marine trips over a full chick rearing period (Additional file 1: Figure S6). The probability of marine foraging trips depended on a triple interaction between chick age, sex of the individual and the day of the week (Table 1), with a highest prevalence of marine trips observed in males and during weekdays. The relative proportion of marine feeding trips decreased with increasing chick age, most sharply for males during weekends (Fig. 1).

**Table 1:** Binomial GLM for the proportion of marine trips, against the interaction between chick age, sex of the individual and period of the week

Dependent variable	Factor		Estimated marginal mean or coefficient ± s.e.	χ^2^_(1)_	*P*
Proportion of marine trips	Chick age			0.6	0.454
	Chick age x sex			0.01	0.929
	Chick age x weekend			5.4	0.020
	Sex	Female		8.2	0.004
		Male			
	Weekend	Working day		36.2	< 0.001
		Weekend			
	Sex x weekend			11.0	0.001
	Female	Working day	0.17 ± 0.06		
		Weekend	0.03 ± 0.01		
	Male	Working day	0.42 ± 0.1		
		Weekend	0.18 ± 0.06		
	Chick age x sex x weekend			5.9	0.015
	Female	Working day	−0.001 ± 0.001		
		Weekend	0.001 ± 0.001		
	Male	Working day	−0.002 ± 0.002		
		Weekend	−0.004 ± 0.003		

Estimate values are back-transformed from the logit scale. Estimated marginal means are the estimated mean for a factor level or a factor level combination. Output of the corresponding analyses of deviance for the significance of the model terms

*s.e.*, standard error

In general, marine trips had higher energy expenditure rates (mean ODBA = 0.63 *g;* SD = 0.16 *g*) and shorter durations (mean = 2.7 h; SD = 1.9 h) compared to terrestrial trips (mean ODBA = 0.45 *g*; SD = 0.14 *g*; mean duration = 4.5 h; SD = 3.2 h). With increasing chick age, foraging trips became less energy intensive, as energy expenditure rates decreased for both marine and terrestrial trips, while trip duration increased substantially for terrestrial trips but only slightly so for marine ones (Table 2; Additional file 1: Figure S7a, c).

**Table 2:** Linear mixed models for the trip energy consumption rate (average ODBA) and trip duration, against chick age and the foraging habitat

Dependent variable	Factor		Estimated marginal mean or coefficient ± s.e.	χ^2^_(1)_	*P*
Average ODBA (*g*)	Chick age		−0.002 ± 0.0004	24.0	< 0.001
	Habitat	Terrestrial	0.43 ± 0.01	505.9	< 0.001
		Marine	0.63 ± 0.01		
Trip duration (h)	Chick age			50.4	< 0.001
	Habitat	Terrestrial	3.93 ± 0.17	32.0	< 0.001
		Marine	2.61 ± 0.23		
	Chick age x habitat	Terrestrial	0.05 ± 0.01	8.7	0.003
		Marine	−0.04 ± 0.01		

Estimated marginal means are the estimated mean for a factor level or a factor level combination. Output of the corresponding analyses of deviance for the significance of the model terms

*s.e.*,standard error

**Fig. 2:**
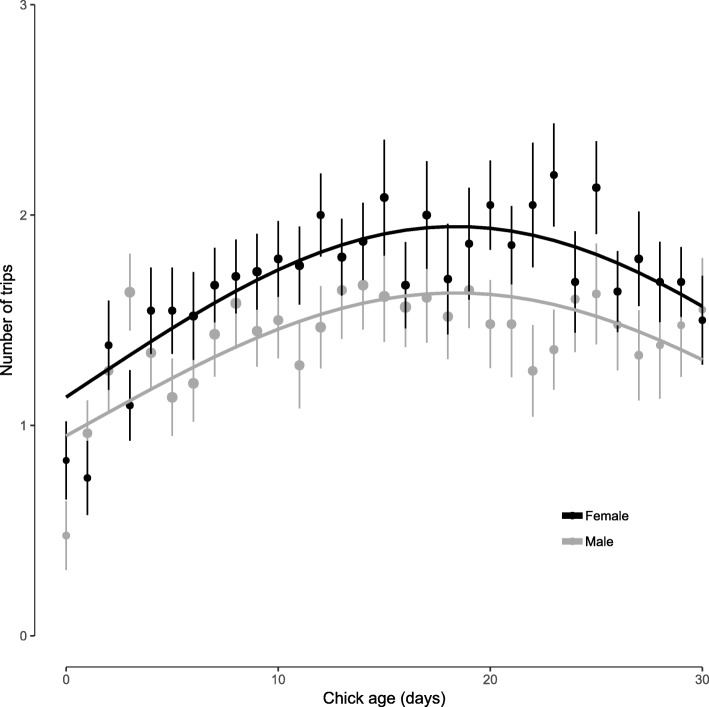
Number of trips performed per individual and day against a second order polynomial of chick age, by sex. Points indicate the mean across individuals, per value of chick age, bars indicate the standard error of the mean. Size of points is proportional to sample size

As chicks became older, individuals initiated an increasing number of foraging trips per day, that reached a peak before 20 days after hatching of the first egg, and subsequently decreased (Fig. 2, χ^2^_(2)_ = 42.13, *p* < 0.001). Throughout these periods, females initiated on average more trips than males (average females = 1.7; SD = 0.3; Average males = 1.4; SD = 0.2; χ^2^_(1)_ = 10.57, *p* = 0.001). The growing number of trips per day, and their increasing duration along the chick rearing period resulted in an increase in the summed daily ODBA and time spent outside of the colony (Table 3). Given the shorter duration of marine trips, days spent foraging only at sea implied less time spent away from the colony, while the differences in summed daily ODBA between fully marine and terrestrial days were statistically significantly different, but fully marine days showed only slightly higher summed ODBA (Additional file 1: Figure S7b, d).

**Table 3:** Zero-inflated Poisson linear model for the number of trips initiated per day against chick age and sex. Linear mixed models for proxies of daily energy and time investments against chick age and the proportion of marine trips

Dependent variable	Factor		Estimated marginal mean or coefficient ± s.e.	χ^2^_(1)_	*P*
Number of trips	Chick age	Days	0.06 ± 0.01	39.9	< 0.01
		Days^2^	−0.002 ± 0.0003	32.7	< 0.01
	Sex	Female	1.90 ± 0.07	18.7	< 0.01
		Male	1.59 ± 0.06		
Summed daily ODBA (*g*)	Intercept		9.47 ± 0.41		
	Chick age		0.09 ± 0.01	38.7	< 0.001
	Proportion of marine trips		0.94 ± 0.36	7.0	0.008
Time away from colony (h)	Intercept		8.76 ± 0.38		
	Chick age		0.14 ± 0.01	113.0	< 0.001
	Proportion of marine trips		−1.95 ± 0.29	46.3	< 0.001

Estimated marginal means are the estimated mean for a factor level or a factor level combination. Output of the corresponding analyses of deviance for the significance of the model terms

*s.e.*,standard error

**Fig. 3:**
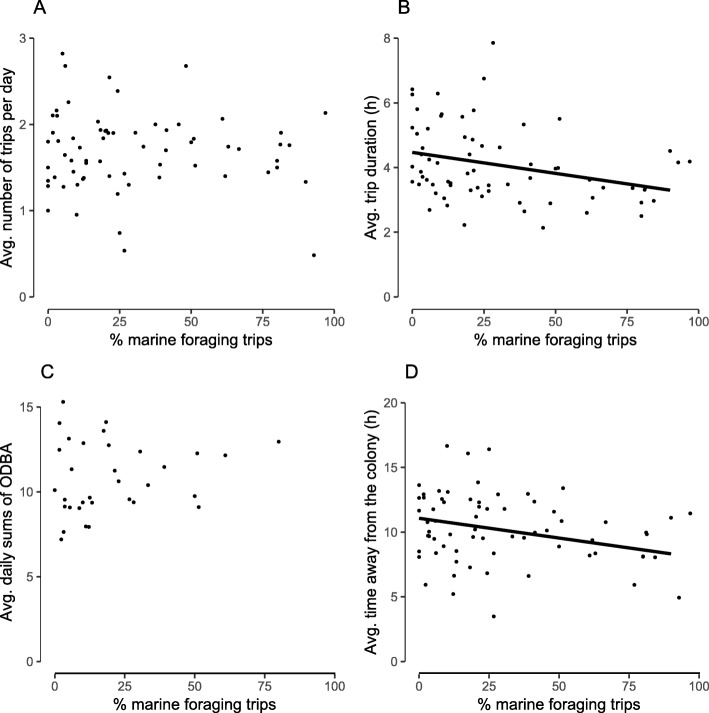
Time and energy costs averaged per individual over a chick rearing period, plotted against the individual’s proportion of marine foraging trips. **a** average number of foraging trips per day, **b** average trip duration, **c** average of the total daily sums of ODBA and **d** average time spent outside of the colony in a day. Regression lines are plotted for the significant relationships

When averaging costs over a complete chick rearing period (Table 4), the mean of the summed daily ODBA varied widely between individuals (average = 9.8 *g*; SD = 3.1 *g*), where the bird with the highest values (mean of summed daily ODBA = 15.3 *g*) spent more than twice the amount of energy estimated for the individual with the lowest value (ODBA = 7.2 *g*). This variation, however, did not relate to the proportion of marine trips over the period, as neither did the mean number of trips initiated (average = 1.7; SD = 0.4; Fig. 2a, c). Consequently, the mean trip duration (average = 4.1 h; SD = 1.2 h) and daily time spent away from the colony (average = 10.3 h; SD = 2.7 h) decreased with increasing reliance on marine foraging (Fig. 3b, d), while the average daily energy expenditure did not.

**Table 4:** Regressions of time and energy investment proxies, averaged per individual over a chick-rearing period, against the overall proportion of trips recorded at sea

Dependent variable	Factor	Estimated marginal mean or coefficient ± s.e.	Statistic	*P*
Avg. N trips per day	Intercept	1.73 ± 0.08		
	Proportion of marine trips	−0.12 ± 0.20	F _(1, 66)_= 0.4	0.54
Avg. trip duration (h)	Intercept	4.47 ± 0.2		
	Proportion of marine trips	−1.3 ± 0.5	F _(1, 66)_= 6.8	0.01
Avg. of summed daily ODBA (*g*)	Intercept	10.48 ± 0.53		
	Proportion of marine trips	1.67 ± 1.88	F _(1, 31)_= 0.8	0.38
Time away from the colony (h)	Intercept	11.07 ± 0.53		
	Proportion of marine trips	−3.05 ± 1.5	F _(1, 51)_= 4.2	0.046

Estimated marginal means are the estimated mean for a factor level or a factor level combination. Output of the corresponding F-tests for the significance of the model terms

*s.e.*,standard error

## Discussion

For Lesser Black-backed Gulls nesting in the colony of Zeebrugge, marine foraging trips typically implied higher energetic costs but lower time investments than terrestrial trips. As chicks grew older, the relative proportion of marine feeding trips decreased, trip durations increased, and energy costs per trip became lower. Trip frequencies increased at the beginning of the chick rearing period, and decreased after 20 days. Over a full chick rearing period, energy costs, as approximated by summed daily ODBA, varied widely among individuals, but no clear differences emerged between terrestrial and marine foragers. In contrast, individuals that relied more often on marine resources spent less time away from the colony on average.

Marine foraging was more frequently observed in males than in females, and during weekdays, while its prevalence tended to decrease with increasing chick age. Similar cases of habitat partitioning between sexes have often been ascribed to competitive differences in relation to body size (e.g. [37, 71]), and earlier studies involving Lesser Black-backed Gulls showed that marine foraging generally implies highly competitive conditions to obtain food at fishing vessels, [54, 72]. However, sex-related variation in foraging strategies may also result from different optimization criteria during foraging (e.g. risk aversion versus reward maximization) ( [73] and references therein), intra-pair competition avoidance or bet-hedging. The effects of fishing activity on marine foraging, as evident from the lower number of marine trips during weekends, are well established for scavenging seabirds [36, 74, 75]. Whether, and to what extent, local gulls that still forage at sea during weekends are relying more on pelagic, naturally occurring prey (e.g. Swimming Crabs Gens. *Liocarcinus*, *Polybius* [48, 76];), remains to be investigated.

Marine trips were more energy demanding per unit of time, as inferred from the higher overall dynamic body acceleration, but shorter in duration. Indeed, earlier studies involving Lesser Black-backed Gulls showed that marine foraging generally implies higher rates of flight [77]. However, the shorter average duration of marine trips and similar number of initiated marine and terrestrial trips, translates into a lack of trends in energy expenditure between marine and terrestrial foragers. This may be interpreted as either the consequence of energetic constraints, whereby marine foragers have to compensate for the greater effort by resting more in or near the colony, or as an optimization of time costs by marine foragers, reducing time constraints in chick guarding. Yet, energetic costs of different foraging strategies may also comprise non-movement related traits, such as temperature regulation, that are not reflected in overall dynamic body acceleration [35]. We assumed that the limited geographical framework and the coincidence in time of the observations for different individuals in our study allow for the energetic comparison of marine versus terrestrial strategies based on ODBA, as environmental conditions would not vary greatly between individuals. Underlying age and size-related differences in metabolic rate, on the other hand, may still play a role in the energy budgets of the tracked birds.

Differences in nest attendance, associated with foraging effort, have elsewhere been linked to differences in breeding success due to brood predation in the co-occurring Herring Gull [78]. Time-efficient strategies might be favored by males in order to improve their capacity to defend the nesting territory, reducing exposure of the brood to predation [39, 79, 80]. Males being larger, it may then be more effective for them to take part in nest defense instead of females [81]. Additionally, as mentioned above, the resulting niche partitioning between nest mates could as well constitute a competition avoidance strategy, or else a bet-hedging strategy, to reduce the chances of unsuccessful foraging by both individuals. However, males and females of this same species have elsewhere been observed to be equally proficient at brood protection during incubation [82], and there is no evidence that intra-pair competition avoidance can constitute an evolutionarily stable strategy in this context, rather than a circumstantial side-effect. These suggestions also do not explain why some males showed virtually no marine foraging. Alternatively, the fact that (i) marine foragers did not use the extra time they dispose of compared to terrestrial foragers in performing more trips, and (ii) increasing chick demand for food was met by initiating more energy-efficient yet time-demanding terrestrial foraging trips, suggests that gulls breeding in Zeebrugge may be energy-constrained, rather than time-constrained, at least in the case of marine foragers. Finally, time spent inside the colony may increase predation risk for the adults themselves, but if this constituted a perceived cost, we would expect the time spent away from the colony to be independent of the trip durations, as they would choose to spend more time elsewhere, even while not foraging. Instead, both time spent away from the colony and trip durations varied similarly in relation to foraging choice, indicating that shorter time investments in foraging translated into enhanced nest attendance.

Apart from time and energy related costs, the yields obtained from each foraging strategy may also determine their relative suitability during chick rearing. Yields obtained can differ in quantity, energy density [83, 84], nutrient content [85] and/or variability in composition [86]. The lower use of marine resources and varying frequency of foraging trips with increasing chick age in our study were likely driven by changing dietary requirements of the brood. Recently hatched chicks generally demand energy- and calcium-rich food such as fish [85, 87], while older chicks require more energy, thus larger amounts of food, which are obtainable at a lower energy cost on land (see higher). In parallel, the vulnerability of gull chicks is highest during the first days after hatching, when the presence of a parent is crucial to chick survival [80]. Time investments in foraging may thus become less costly with advancing chick age.

The observed differences in energy and time costs between marine and terrestrial foraging should be taken into account when assessing the potential responses of gull populations to environmental change, which involves short-term changes in human behavior, such as the European ban on discards, implemented from 2019 onwards [88], as well as climate change. Since marine foraging is energetically more costly than its terrestrial counterpart, it may be more vulnerable to changes in environmental factors that affect foraging energetics, such as wind conditions. Conversely, the more time demanding terrestrial foraging may be more sensitive to factors that affect search, wait and handling times, such as human behavior or environmental conditions that affect the accessibility of food. These factors could be partly responsible for the large unexplained variation in time and energy costs within habitats reported here, both at the daily scale and over the full chick rearing period. In this context, characterizing individual responses to day-to-day environmental variability may help further elucidate the sources of variation in parental energy and time investment during chick rearing.

## Conclusions

Marine foraging, favored by males and during working days, implies larger energy expenditure rates and shorter time investments than terrestrial foraging in Lesser Black-backed Gulls. However, at a scale of several days, a greater reliance on marine foraging does not result in larger total energy expenditures, while it does demand lower total time investments in foraging. Early chick growth drives an increase in foraging effort, as well as a gradual shift toward more terrestrial foraging. Given these trends, changes in food availability at the marine or terrestrial habitats may have different effects between sexes, and along the various stages of chick growth.

## Supplementary information

**Additional file 1: **Contains a map of the study area with location data plotted (**Figure S1**), a summary of sample sizes (**Table S1**; **Figures S2–S4**), an assessment of ODBA as a good indicator of energy expenditure, through its relationship with the amount of flapping flight (**Figure S5**), a boxplot for the distribution of the proportion of marine foraging trips per individual, by sex (**Figure S6**), plots for the energy and time investments calculated per trip and day, against chick age, including regression lines for significant relationships (**Figure S7**), and model diagnostic plots (**Figures S8–S11**).

**Additional file 2.** Contains the complete R script necessary to (1) derive the datasets used for all statistical analyses in the present study from the GPS positions and accelerometer data, (2) run the analyses and (3) produce the figures.

## Data Availability

The dataset analyzed during the current study is available on Movebank, under study name LBBG_ZEEBRUGGE. URL: https://www.movebank.org/panel_embedded_movebank_webapp?gwt_fragment=page=studies, path = study985143423.

## Abbreviations

GLMM: Generalized Linear Mixed Model
GPS: Global Positioning System
ODBA: Overall Dynamic Body Acceleration
SD: Standard Deviation
UvA-BiTS: University of Amsterdam Bird Tracking System
