# Supplementary material for: Time and energy costs of different foraging choices in an avian generalist species
Source: Mov Ecol. 2019 Dec 30;7:41. doi: 10.1186/s40462-019-0188-y (PMC6937837; doi:10.1186/s40462-019-0188-y)
Supplement: Supplementary file 1 — Additional file 1: Contains a map of the study area with location data plotted (Figure S1), a summary of sample sizes (Table S1; Figures S2–S4), an assessment of ODBA as a good indicator of energy expenditure, through its relationship with the amount of flapping flight (Figure S5), a boxplot for the distribution of the proportion of marine foraging trips per individual, by sex (Figure S6), plots for the energy and time investments calculated per trip and day, against chick age, including regression lines for significant relationships (Figure S7), and model diagnostic plots (Figures S8–S11). [file 40462_2019_188_MOESM1_ESM.docx]

**Study area:**


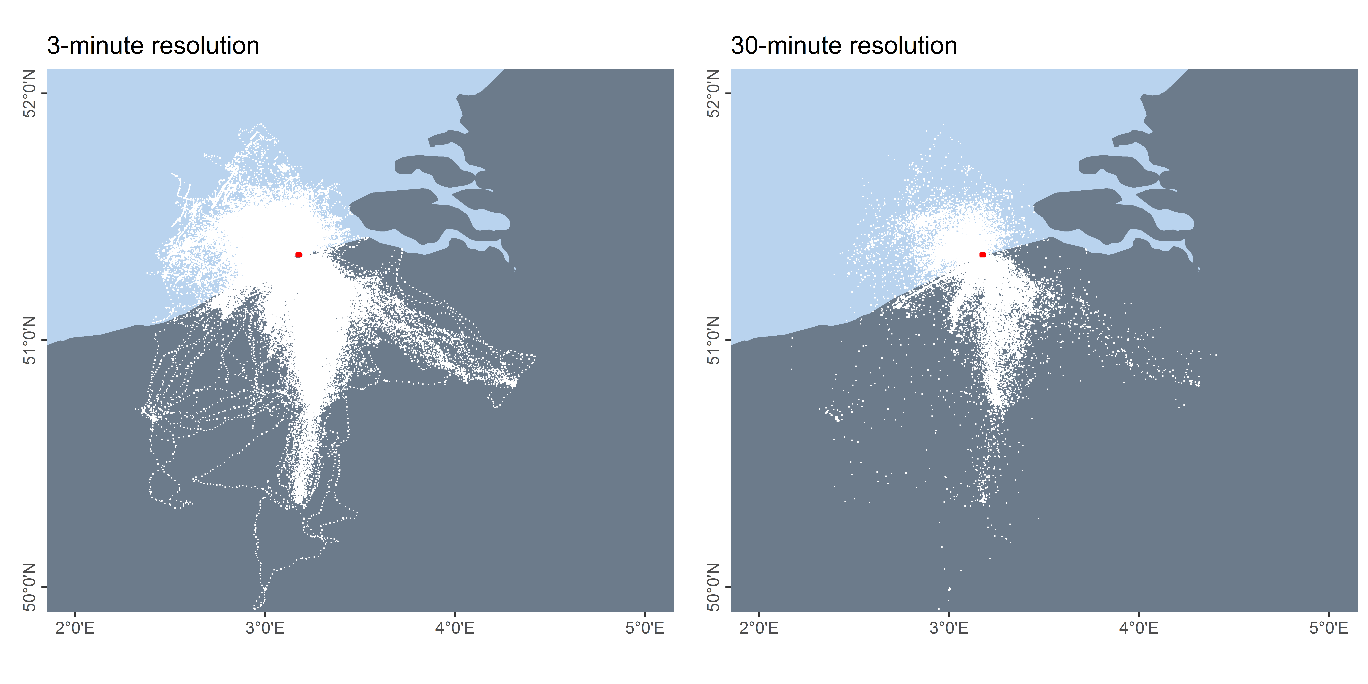


*Figure S1 Recorded positions of tracked birds, at a 3-minute (left) and 30-minute resolution (right). The area of the colony beyond which a trip is considered to take place is indicated in red.*

**Sample size:**

*Table S1: Sample size per year. Number of individuals newly equipped with a GPS transmitter (N_0_), number of individuals-year included in analyses (N_e_), and number of individuals-year with accelerometer data (N_acc_).*

|  | N_0_ | N_e_ | N_acc_ |
| --- | --- | --- | --- |
| 2013 | 22 | 12 | 0 |
| 2014 | 21 | 16 | 0 |
| 2015 | 12 | 12 | 10 |
| 2016 | 11 | 14 | 14 |
| 2017 | 4 | 6 | 6 |
| 2018 | 5 | 6 | 3 |
| Total | 75 | 68 | 33 |


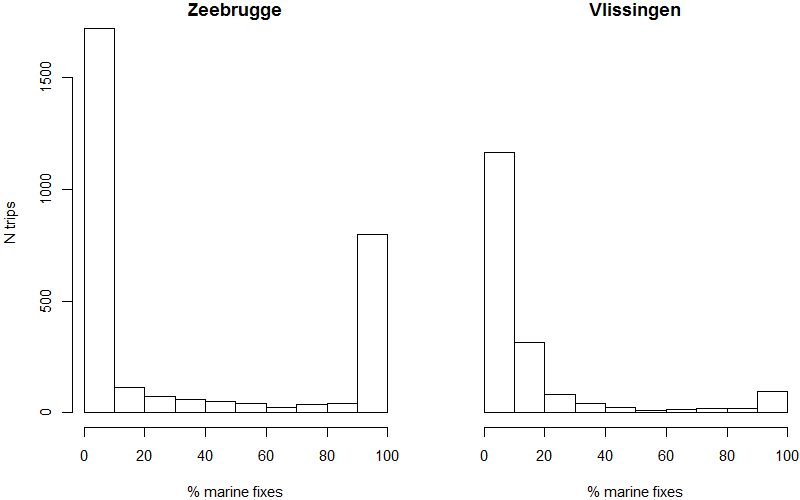


Figure S2Histogram for the percentage of total fixes recorded in the marine habitat per bout for all tracked individuals, by colony. The majority of foraging trips involve either >90% terrestrial fixes or >90% marine fixes, particularly in Zeebrugge.


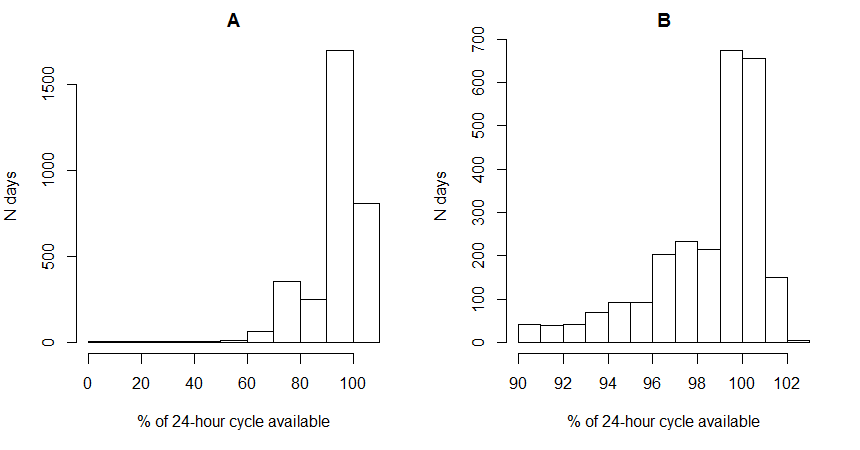


Figure S3 Histogram for the percent of a full 24-hour cycle available per combination of date and individual in (A) the original sample, and (B) after subsetting for at least 90% of 24 hours available.


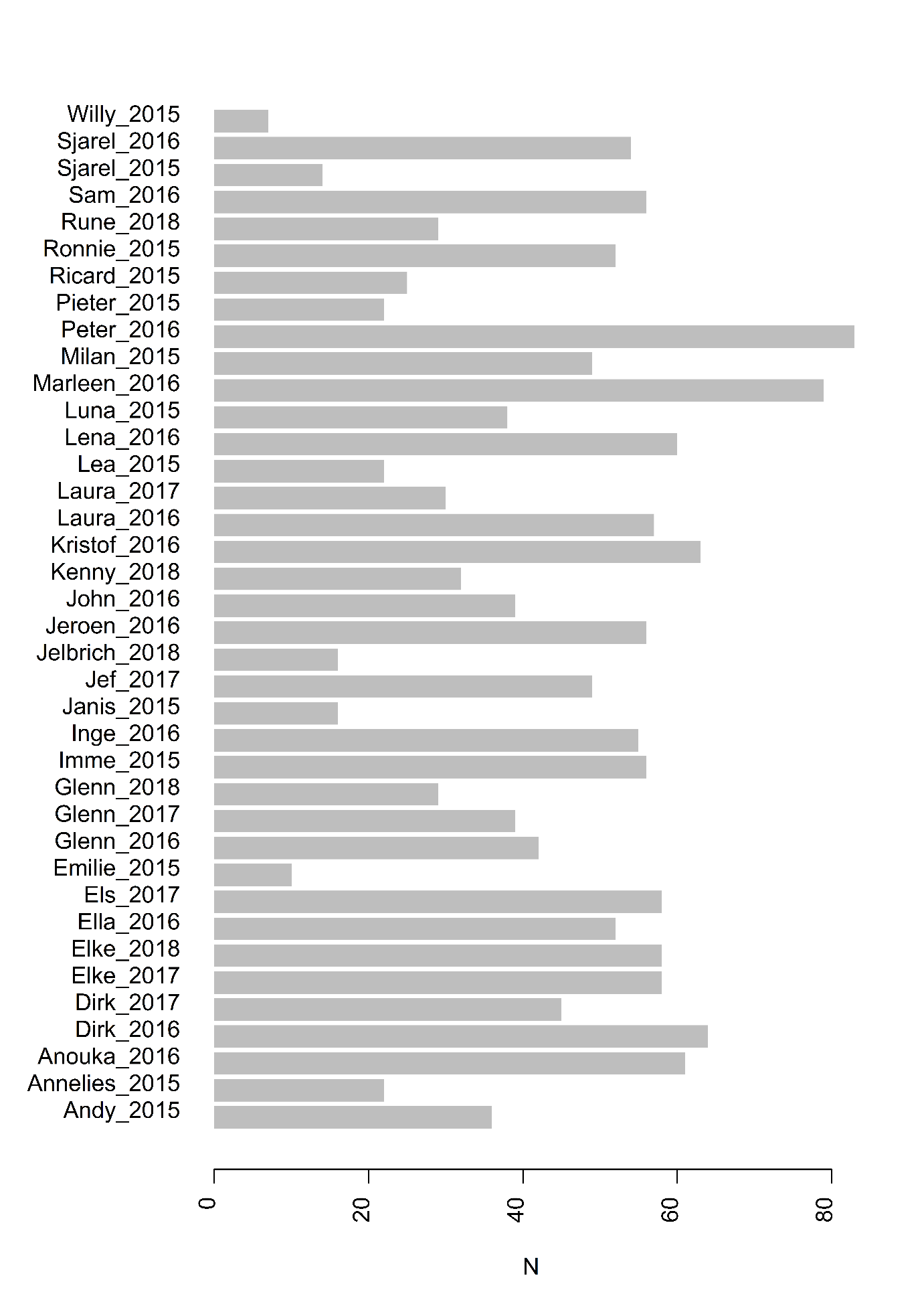


Figure S4 Number of trips per individual and year used in the analyses of the variation in ODBA (at least 90% of fixes with accelerometer measurements).

**Assessment of ODBA:**


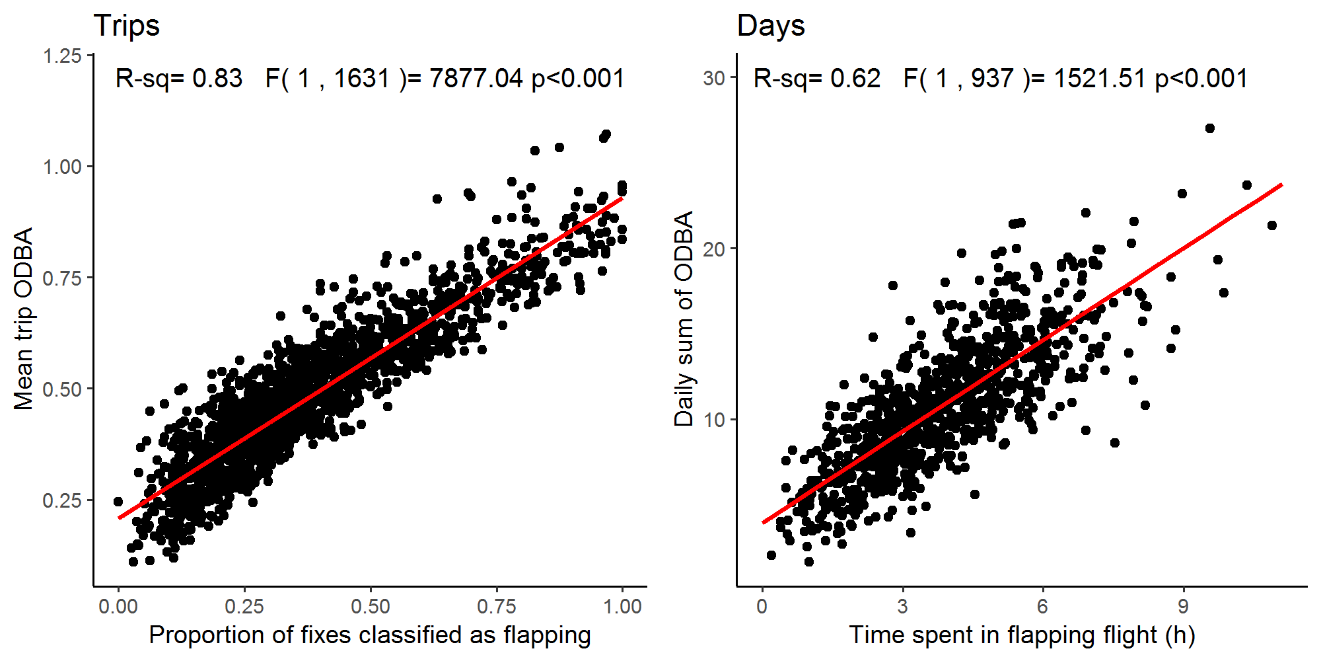


Figure S5 Linear regressions for the mean trip ODBA against the proportion of fixes classified as flapping per trip (left) and daily sum of ODBA against the daily time spent in flapping flight (right). Classification of flapping fixes was performed for all records where accelerometer data was available, using a random forest classifier as in (77).

**Distribution of marine foraging:**


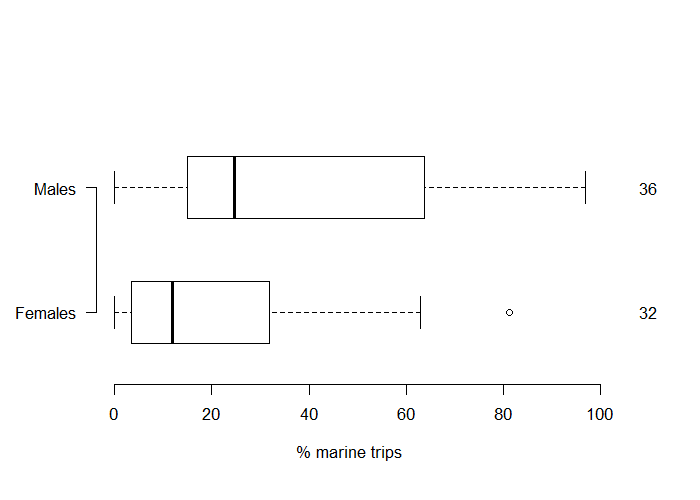


Figure S6 Proportion of fully marine foraging trips performed by individuals over a full chick rearing period, by sex.

**Trip and day-based cost proxies:**

Table S2 Estimated marginal means and results of likelihood ratio tests for the effect of sex in trip and day-based analyses of costs.

| Scale | Dependent variable |  | Sex | Estimated marginal mean ± s.e. | χ^2^_(1)_ | P |
| --- | --- | --- | --- | --- | --- | --- |
| Trips | Mean trip ODBA (*g*) | Terrestrial | Females | 0.43 ± 0.37 | 0.05 | 0.82 |
|  |  |  | Males | 0.43 ± 0.38 |  |  |
|  |  | Marine | Females | 0.63 ± 0.57 |  |  |
|  |  |  | Males | 0.63 ± 0.58 |  |  |
|  | Total trip duration (h) | Terrestrial | Females | 4.72 ± 0.20 | 0.11 | 0.74 |
|  |  |  | Males | 4.62 ± 0.20 |  |  |
|  |  | Marine | Females | 2.84 ± 0.23 |  |  |
|  |  |  | Males | 2.75 ± 0.21 |  |  |
| Days | Total daily ODBA (*g*) | | Females | 11.3 ± 0.49 | 0.71 | 0.40 |
|  |  |  | Males | 10.7 ± 0.47 |  |  |
|  | Time away from the colony (h) | | Females | 10.4 ± 0.44 | 0.04 | 0.84 |
|  |  |  | Males | 10.3 ± 0.43 |  |  |


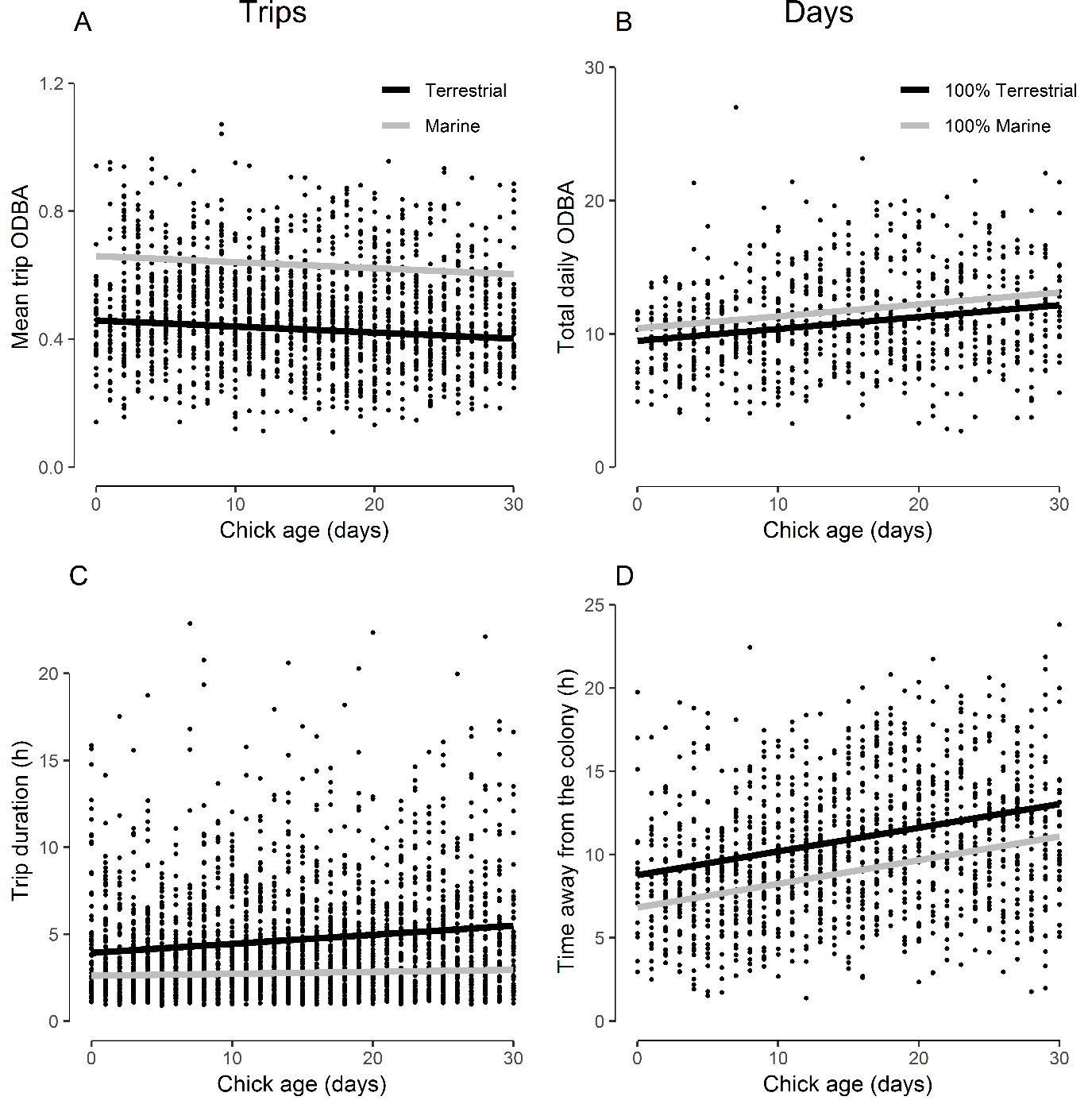


Figure S7: Energy and time investments calculated per trip (left) and per day (right) by habitat (marine or terrestrial) against chick age in birds nesting in Zeebrugge: (A) Mean trip ODBA (5-minute resolution). (B) Daily sums of ODBA (30-minute resolution). (C) Trip duration. (D) Daily time spent outside the colony. Regression lines are plotted for significant relationships.

**Model diagnostics**


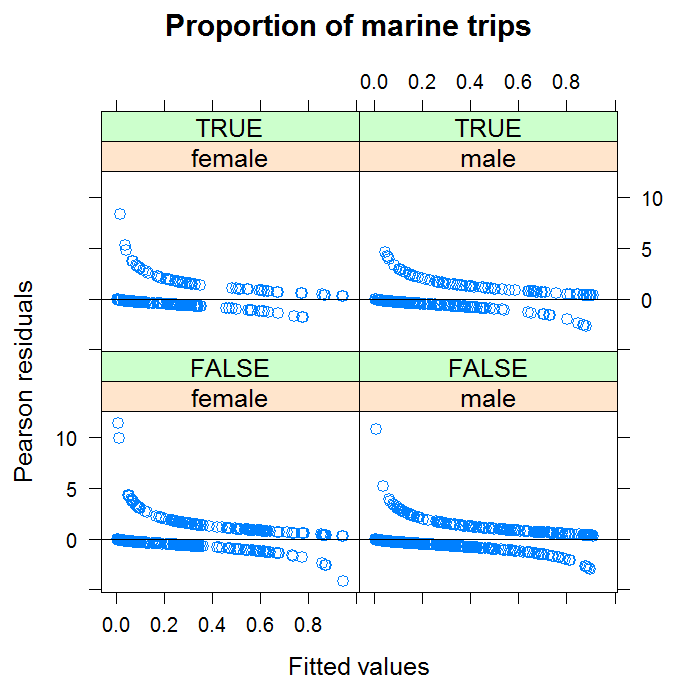


Figure S8 Pearson residuals against fitted values of the binomial generalized linear mixed model for the proportion of marine trips initiated. TRUE = weekend; FALSE = weekday.


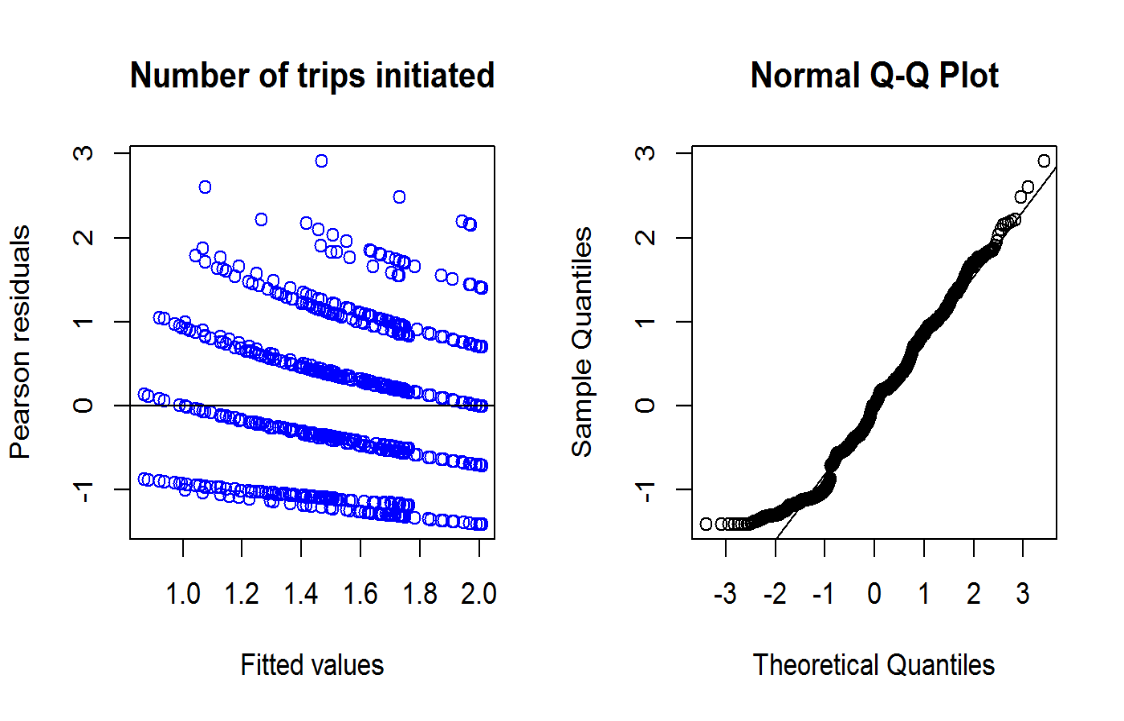


Figure S9 Pearson residuals against fitted values, and Q-Q plot of Pearson residuals of the zero-inflated Poisson regression for the number of trips initiated against chick age.


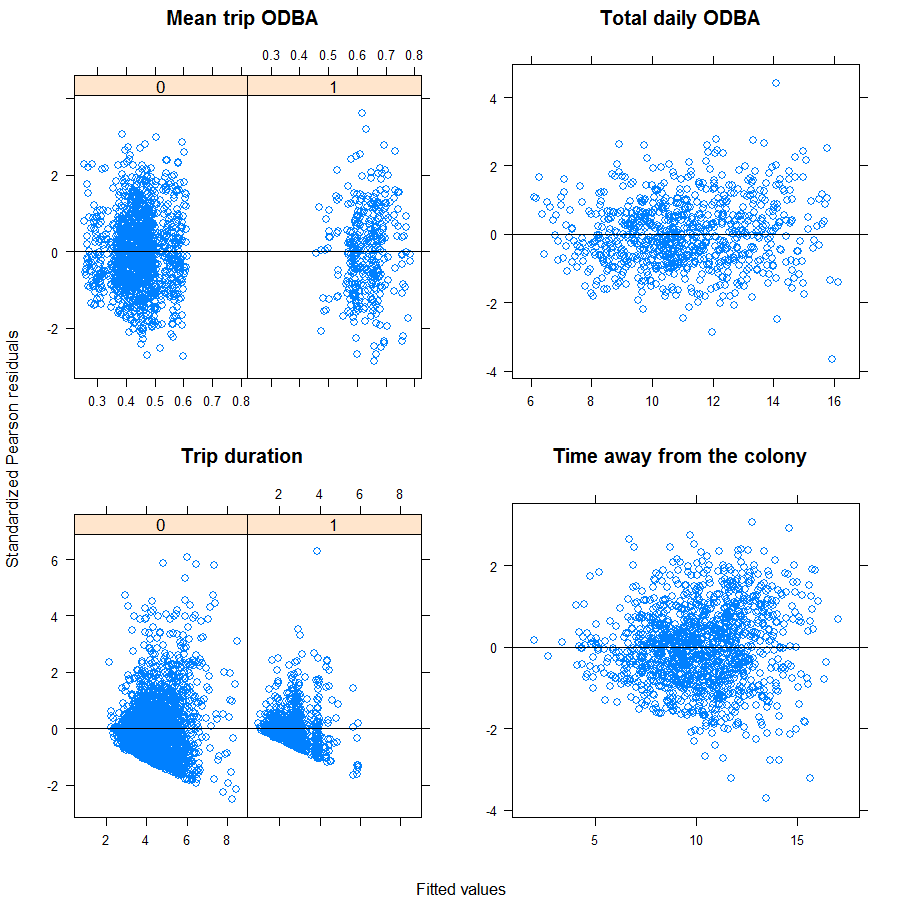


Figure S10 Pearson residuals against fitted values of the linear mixed models for the time and energy costs of foraging trips (LEFT) and daily time and energy investments (RIGHT). 1 = marine trip; 0 = terrestrial trip


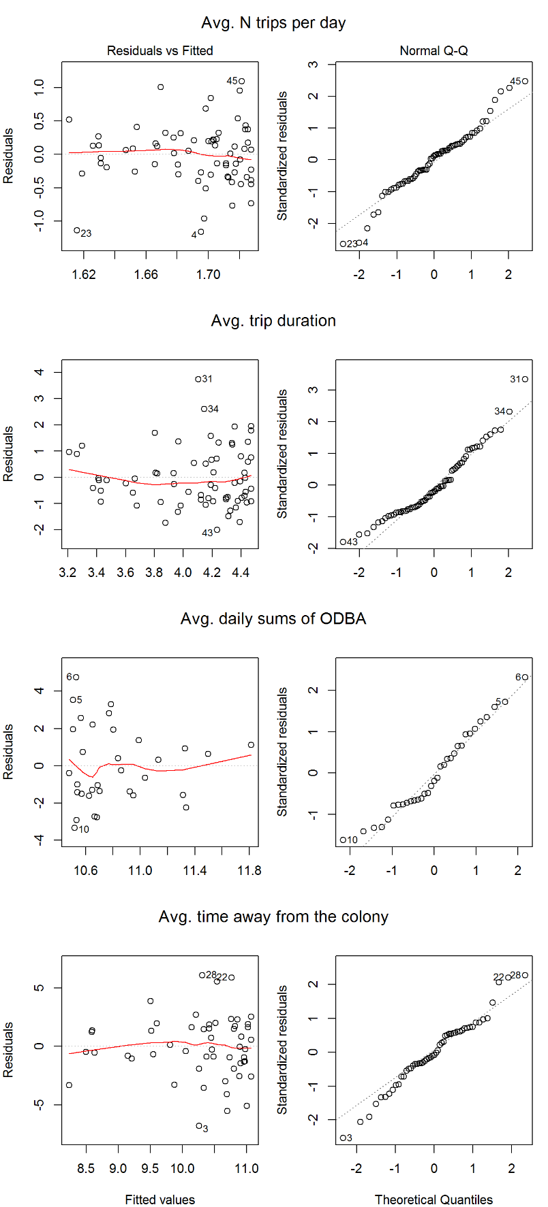


Figure S11 Residuals against fitted values and Q-Q plot of residuals for the linear models of time and energy costs averaged per individual over a chick rearing period, against the individual’s proportion of marine foraging trips
